# Expression of hepatic Fibroblast Growth Factor 19 is enhanced in Primary Biliary Cirrhosis and correlates with severity of the disease

**DOI:** 10.1038/srep13462

**Published:** 2015-08-21

**Authors:** Ewa Wunsch, Małgorzata Milkiewicz, Urszula Wasik, Jocelyn Trottier, Agnieszka Kempińska-Podhorodecka, Elwyn Elias, Olivier Barbier, Piotr Milkiewicz

**Affiliations:** 1 Department of Clinical and Molecular Biochemistry, Pomeranian Medical University in Szczecin, Szczecin, Poland; 2 Department of Medical Biology, Pomeranian Medical University in Szczecin, Szczecin, Poland; 3 Laboratory of Molecular Pharmacology, CHU-de-Québec & Faculty of Pharmacy, Laval University, Québec, QC, Canada; 4 University of Birmingham, Birmingham, United Kingdom; 5 Liver and Internal Medicine Unit, Department of General, Transplant and Liver Surgery of the Medical University of Warsaw, Warsaw, Poland

## Abstract

Cholestasis induces adaptive mechanisms protecting the liver against bile acids (BA) toxicity including modulation of BA synthesis. Whether fibroblast growth factor 19 (FGF19) or farnesoid X receptor (FXR) dependent signaling are involved in the regulation of BA homeostasis in primary biliary cirrhosis (PBC) remains unknown. Here we analyzed hepatic expression of FGF19 and other genes relevant to the adaptive response to cholestasis in tissues from non-cirrhotic (n = 24) and cirrhotic (n = 21) patients along with control tissues (n = 21). Moreover we searched for relationships between serum FGF19 and laboratory/clinical findings in 51 patients. Hepatic FGF19 mRNA expression was increased in non-cirrhotic and cirrhotic tissues (9-fold,*p* = 0.01; 69-fold,*p* < 0.0001, respectively). Protein levels of FGF19, FGF receptor 4, FXR and short heterodimer partner were increased in cirrhotic livers (9-fold, *p* < 0.001; 3.5-fold,*p* = 0.007; 2.4-fold,*p* < 0.0001; 2.8-fold,*p* < 0.0001 *vs* controls, respectively) which was accompanied by down-regulation of CYP7A1 (50% reduction, *p* = 0.006). Serum and liver levels of FGF19 correlated with worse liver biochemistry, BAs, quality of life and Mayo Risk Score. Serum FGF19 was elevated in UDCA non-responders. We conclude that PBC induces characteristic changes in liver expression of BAs synthesis regulatory molecules. FGF19 correlates with severity of liver disease and can potentially serve as an indicator of chronic cholestatic liver injury.

Primary biliary cirrhosis (PBC) is a slowly progressive autoimmune condition of unknown etiology, predominately affecting middle-aged women^1^. It is associated with pruritus, chronic fatigue and cognitive decline^2^,^3^. Antimitochondrial autoantibodies are serological hallmarks of this condition^4^. The disease selectively affects small intrahepatic bile ducts, leading to hepatocellular accumulation of bile salts, chronic cholestatic injury of the liver, and biliary cirrhosis in a proportion of patients. *In vitro* and animal studies have shown that cholestasis of any origin may induce adaptive mechanisms which protect the liver against bile salt toxicity^5^,^6^. One of these is a suppression of bile acids synthesis via inhibition of expression of a rate-limiting enzyme, cholesterol 7α-hydroxylase (CYP7A1)^7^,^8^. It is well established that farnesoid X receptor (FXR) is essential for negative feedback regulation of CYP7A1. FXR is expressed in the liver and small intestine, where it is activated by binding of bile acids. One major target of hepatic FXR is the small heterodimer partner (SHP), which inhibits transcription factor liver receptor homolog-1 (LRH-1), a known positive regulator of CYP7A1. Thus, activation of SHP expression by FXR results in inhibition of transcription of the CYP7A1 gene.

The main regulatory mechanism of bile acids synthesis also involves gut-liver signaling pathway through the polypeptide hormone fibroblast growth factor 19 (FGF19)^9^. FGF19 and its murine ortholog Fgf15 are the founding members of the endocrine FGF subfamily that influences several physiological processes^10^,^11^,^12^,^13^. FGF19 is mainly expressed in the ileum in response to luminal bile salt loading in the post-prandial state in humans. Transcription of FGF19 gene is initiated by bile acids-induced activation of FXR^14^. FGF19 is secreted into portal blood and acts as an endocrine factor which binds to hepatic FGF receptor 4 (FGFR4)/Klotho-β cell-surface receptor complex. The activated FGFR4 receptor suppresses CYP7A1 expression via two independent ways. These include either initiation of extracellular signal-regulated kinase (ERK) signaling pathway^15^ which results in stabilization of SHP protein by inhibition of its proteasomal degradation or via direct blocking of CYP7A1 gene expression through the interaction between c-Jun and transcriptional coactivator PGC-1α^15^,^16^,^17^.

In the ileum, the expression of FXR transcriptionally stimulates FGF19, whereas in the liver FXR initiates transcription of various genes that decrease the concentration of bile acids within the hepatocyte, i.e. reduce the expression of CYP7A1 by increasing SHP expression^8^,^18^. Therefore, hepatic and intestinal FXR act synergistically with FGF19 to downregulate bile acids synthesis in the liver. Although under normal conditions FGF19 expression is virtually absent in human liver, recent studies suggest that in extrahepatic cholestasis FGF19 may be produced also in human hepatocytes^8^,^19^,^20^.

In view of these observations, it may be anticipated that a disturbance in bile acid equilibrium in chronic cholestatic conditions such as PBC would be associated with changes in FGF19 expression. Therefore we analyzed the expression of FGF19 and other genes involved in the regulation of bile acids synthesis in livers of patients with PBC in order to determine whether the FGF19 signaling pathway is activated in PBC. These phenomena are of potential clinical relevance in view of the recent interest in both FXR and FGF19 agonists as potential therapeutic modalities in chronic liver diseases. In order to get more insight in these important processes we also evaluated FGF19 concentration in the serum of PBC patients and searched for their potential relationships with vital clinical and laboratory findings.

## Results

### Group 1 – analysis of FGF-19 in serum

**Figure 1:**
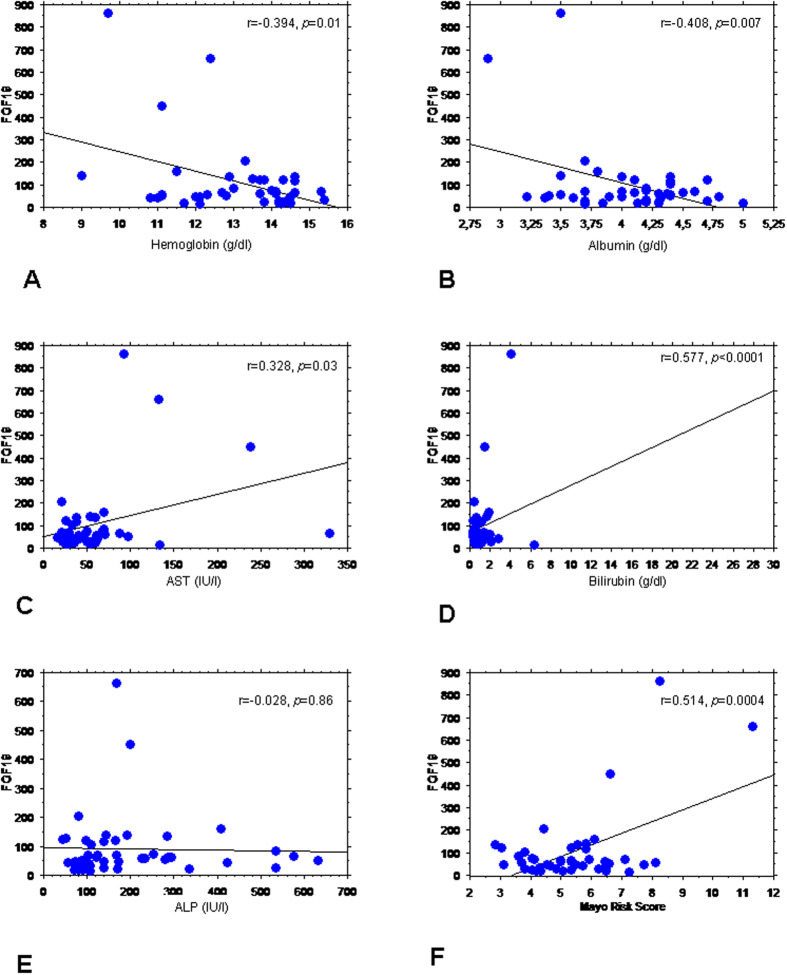
Correlation between serum FGF19 (pg/ml), laboratory parameters and Mayo Risk Score. FGF19 serum concentration correlated with several laboratory parameters: (**A**) hemoglobin, (**B**) albumin, (**C**) AST and (**D**) total bilirubin. No correlation between serum FGF19 levels and (**E**) ALP was seen. (**F**) There was significant relationship between FGF19 concentrations and Mayo Risk Score.

Serum levels of FGF19 were unrelated to age, gender and presence of cirrhosis (data not shown). The univariate analysis showed the significant correlation between the serum FGF19 and several laboratory parameters such as: hemoglobin (r = −0.394, *p* = 0.01), albumin (r = −0.408, *p* = 0.007), total bilirubin (r = 0.577, *p* < 0.0001) and AST (r = 0.328, *p* = 0.03). In the multivariate analysis, total bilirubin and hemoglobin were independent variables (*p* < 0.0001). There was a significant relationship between FGF19 concentrations and Mayo Risk Score for PBC (r = 0.514, *p* = 0.0004). These data are shown in Fig. 1. FGF19 correlated with total bile acid concentration and glycine and taurine conjugates of cholic acid (CA), chenodeoxycholic acid (CDCA) and ursodeoxycholic acid (UDCA). It also correlated with 3-O-glucuronide conjugates of CDCA and lithocholic acid (LCA) (Table 1). In the multivariate analysis, glycine species of CDCA, glycine and taurine conjugate of UDCA and finally 3-O-glucuronide of LCA were independent variables related to serum FGF-19 levels (*p* < 0.0001).

Serum FGF19 showed no relationship with health related quality of life (HRQoL) parameters except the borderline correlation with SF-36 *Bodily Pain* domain (r = 0.296, *p* = 0.05) and PBC-40 *Other Symptoms* domain (r = −0.277, *p* = 0.06) and were significantly lower in UDCA responders than in UDCA non-responders (67.5 ± 42.9 *vs*. 167.0 ± 240.3 pg/ml; *p* = 0.04, Table 2).

### Group 2 - analysis of FGF19 in liver tissue

**Figure 2:**
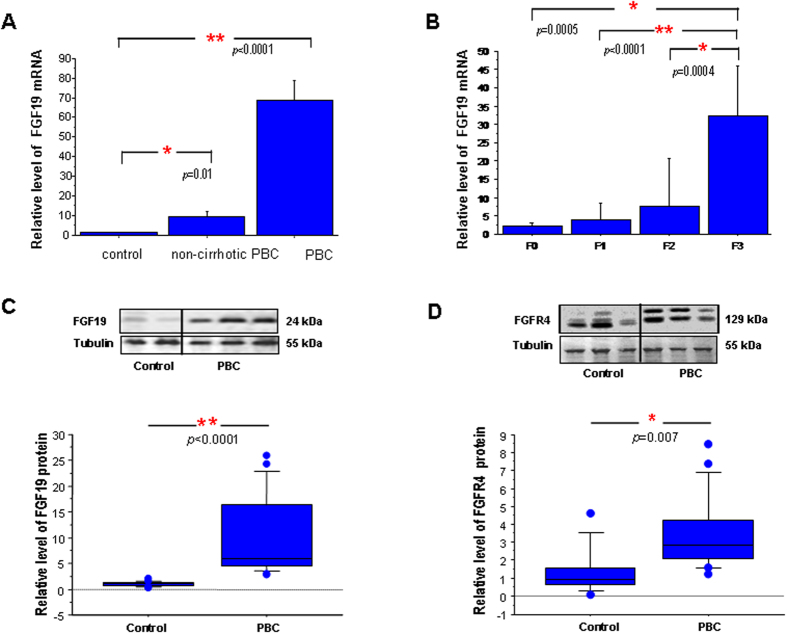
Expression of FGF19 and FGFR4 in liver tissue of patients with PBC. (**A**) FGF19 mRNA expression was significantly enhanced both in non-cirrhotic (n = 24) and cirrhotic (n = 21) PBC livers. (**B**) Expression of liver FGF19 mRNA in non-cirrhotic patients increased along with the stage of fibrosis. Levels of mRNA expression were normalized with glyceraldehyde 3-phosphate dehydrogenase (GAPDH) and presented as a fold-change relative to control. Bars indicate the mean ± SEM. In cirrhotic liver (n = 21) protein levels of (**C**) FGF19 and (**D**) FGFR4 were substantially increased when compared to control tissue (n = 21). Changes in protein levels were determined by densitometry analyses after normalization to α/β tubulin as a control for loading. Data presented as the box-and-whisker plot with median value (middle line).

In non-cirrhotic PBC livers FGF19 mRNA expression was significantly increased in comparison to controls (9 fold; *p* = 0.01, Fig. 2A) and was associated with fibrosis stage at liver biopsy (Fig. 2B). FGF19 mRNA showed a positive correlation with cholestatic parameters (ALP: *Rho* = 0.469, *p* = 0.03; bilirubin: *Rho* = 0.561, *p* = 0.007) and Mayo Risk Score for PBC (r = 0.610, *p* = 0.002). In non-cirrhotic PBC FGF19 mRNA correlated negatively with HRQoL parameters in PBC-27 (*Dryness*: r = 0.519, *p* = 0.02) and SF-36 (*Bodily Pain*: r = −0.531, *p* = 0.02, *General Health*: r = −478, *p* = 0.03 and both *Physical* and *Mental Summary Components*: (r = −0.498, *p* = 0.03 and r = −0.446, *p* < 0.05 respectively). These data are summarized in Table 3. There was no relationship between FGF19 mRNA and age of the patients or UDCA medication (data not shown).

**Figure 3:**
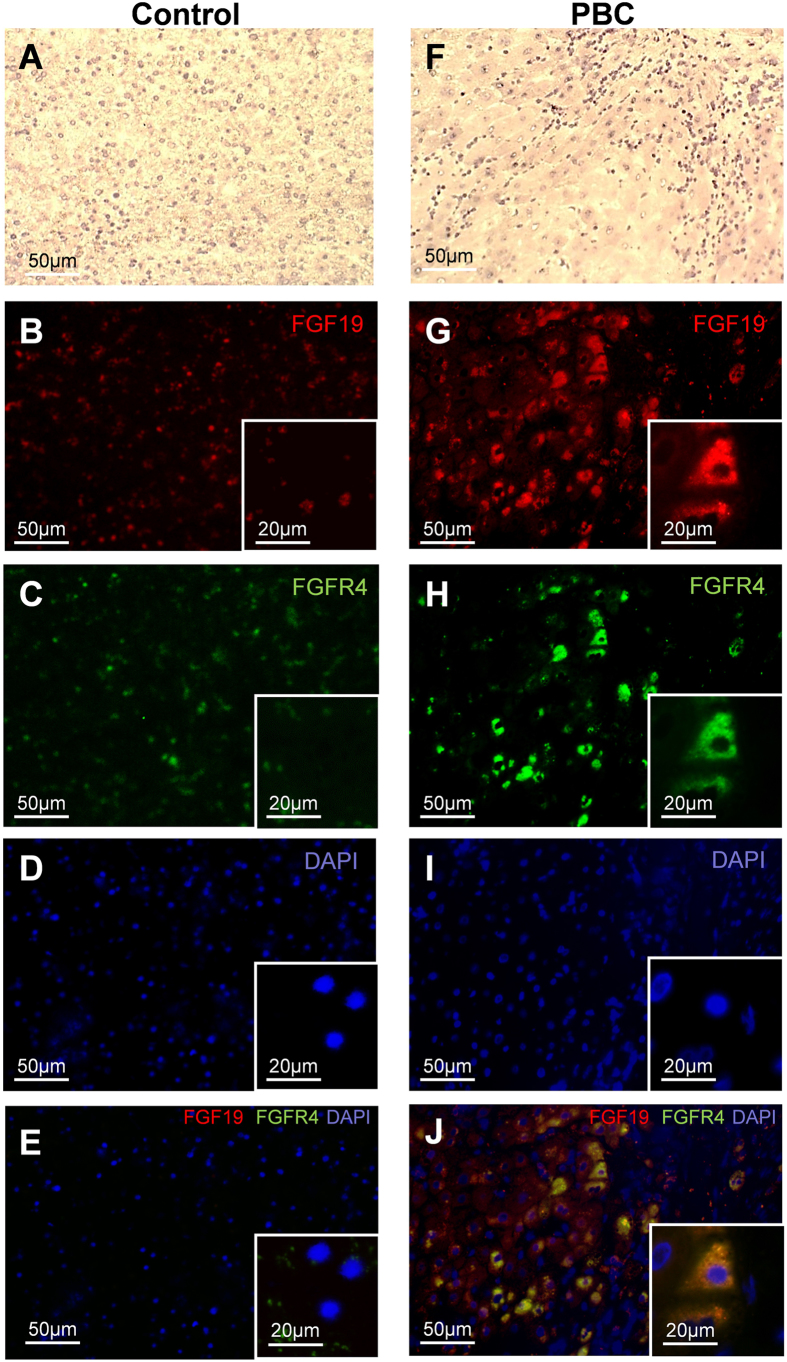
The hepatic expression of FGF19 and FGFR4 protein in cirrhotic patients with PBC and controls. Representative light micrographs of hematoxylin stained liver sections of control (**A**) and PBC (**F**). Immunofluorescence staining of liver tissue showed that in comparison to controls (**B,C**) expression of both (**G**) FGF19 (red) and (**H**) FGFR4 (green) were substantively increased in PBC. Nuclei (blue) stained with DAPI (**D,I**). Merged immunofluorescent images of FGF19, FGFR4 and DAPI (**E,J**) demonstrated that in chronic cholestatic PBC livers FGF19 produced by hepatocytes binds to the FGFR4 (**J**).

In PBC cirrhotic livers FGF19 expression was significantly increased in comparison to controls both at mRNA (69-fold, *p* < 0.0001, Fig. 2A) and protein levels (9-fold, *p* < 0.0001, Fig. 2C) which was accompanied by enhanced expression of FGFR4 protein (3.5 fold, *p* = 0.007, Fig. 2D). Immunohistological analyses depicted a clear difference in hepatic expression of FGF19 and FGFR4 protein between patients with PBC and controls (Fig. 3). Co-localization of the imunostainings demonstrated that FGF19 produced by hepatocytes binds to the FGFR4, which may confirm autocrine/paracrine manner of FGF19 action.

**Figure 4:**
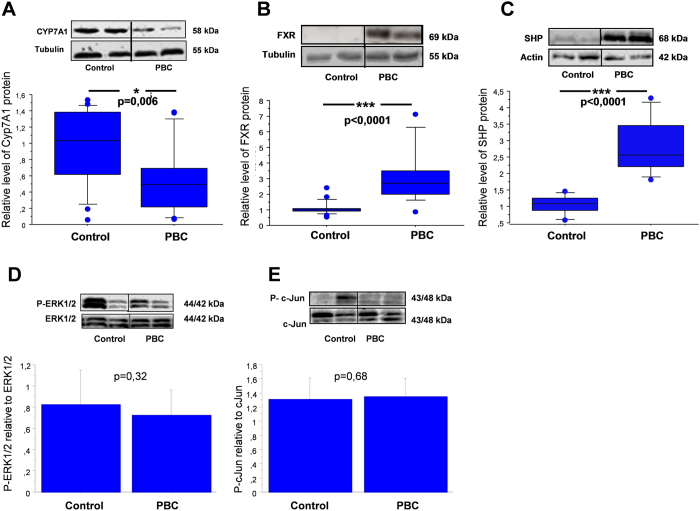
Expression of CYP7A1, FXR, SHP, phosphorylated ERK1/2 and phosphorylated c-Jun proteins in liver tissue of cirrhotic patients with PBC and controls. Changes in (**A**) CYP7A1, (**B**) FXR and (**C**) SHP levels were determined by densitometry analyses after normalization to α/β tubulin or β-actin as a control for loading. Data presented as the box-and-whisker plot with median value (middle line). Phosphorylated (**D**) P-ERK1/2 and (**E**) P-c-Jun bands were quantified by densitometry and normalized to the density of the total ERK1/2 and c-Jun, respectively. Bars indicate the mean ± SEM.

Down-regulation of CYP7A1 under intrahepatic cholestatic conditions was detected (almost 50% reduction, *p* = 0.006 *vs* control, Fig. 4A). In order to investigate the signaling pathway involved in the observed downregulation of CYP7A1, we examined the expression of proteins which were reported to modulate the expression of this enzyme. In cirrhotic livers, FXR and SHP protein levels were significantly increased in comparison to controls (2.4 fold, p < 0.0001 and 2.8 fold , p < 0.0001, respectively, Fig. 4B,C). Neither the ratio of P-ERK1/2 to total ERK1/2 (0.73 ± 0.23 vs 0.83 ± 0.3 in controls), nor the ratio of P-c-Jun to total c-Jun was altered (1,32 ± 0,3 vs. 1,35 ± 0,35 in controls Fig. 4D,E).

## Discussion

Several important and novel findings of potential clinical relevance were identified in this study. Firstly, we demonstrated that expression of FGF19 is strongly induced in livers of patients with PBC as both mRNA and protein levels of FGF19 were remarkably increased in cholestatic livers. Of note, this growth factor was hardly detected in control specimens. It is noteworthy observation since FGF19 expression has not been studied in patients with PBC before and until recently the ileum was believed to be the only source of circulating Fgf15/FGF19, which reaches the liver with portal blood and acts in an endocrine manner^21^. Up till now most of the studies were done on animal models or human cultured hepatocytes. Inagaki *et al.* were unable to identify Fgf15 in sera and livers of mice treated with synthetic FXR agonist GW4064 or CA. These authors reported that Fgf15 was present only in ileum while FGFR4 was highly expressed in mice livers^7^. Studies on cultured human hepatocytes have demonstrated that even though FGF19 is virtually absent in primary, not-activated cells, its expression may be strongly induced after exposure to either GW4064 or CDCA in dose-dependent manner^8^,^20^. In contrast to our studies, some researchers failed to detect signals of FGF19 mRNA from human liver^21^,^22^. However, Schaap *et al.* detected enhanced hepatic FGF19 transcripts in a group of patients with extrahepatic cholestasis caused by a pancreatic tumor^19^. Moreover, these authors reported decrease of this enhancement in response to the restoration of biliary drainage after application of biliary stent, yet remaining higher than in controls^19^. This may suggest that increased liver expression of FGF19 is not restricted to PBC but represents a more universal mechanism in response to cholestasis. On the other hand, in extrahepatic cholestasis, Schaap and colleagues did not observe changes in hepatic FXR or SHP expression, phenomena seen in our study, which implies an activation of a different protective mechanism in sustained intra-hepatic versus acute extra-hepatic cholestasis.

FGF19 is believed to be an endocrine factor that acts on target cells through its cell-surface receptor FGFR4. FGF19 has unique specificity for FGFR4, mediating almost all of FGF19 activities. Thus, in normal conditions, FGF19 primary produced in the ileum influences the bile acids synthesis in the liver. Our study showed that, in prolonged cholestasis, hepatocytes are an important source of FGF19. It is intriguing whether FGF19 affects bile acids homeostasis only in an endocrine manner. Most probably, FGF19 produced in the hepatocyte is then excreted from the cell and acts in an auto- or paracrine manner through its receptor. Indeed, in our study we have observed a significantly increased level of hepatic FGFR4 which was expressed on the same hepatocytes that produced FGF19 (Fig. 3).

The observation of an enhanced expression of FGF19 in cholestatic diseases may raise the question whether a pharmacological activation of FGF19 would be beneficial in these disorders and could offer a potentially promising therapeutic target. Indeed, recent study showed that induction of Fgf15 expression by activation of intestinal FXR transcription protected liver from cholestasis along with reducing hepatic pool of bile acids^22^ and Fgf15/FGF19 mediate liver regeneration in mice^23^,^24^. In contrast, it has been demonstrated that Fgf15/FGF19 is overexpressed in patients with hepatocellular carcinoma, correlates with tumor progression/poor prognosis and exerts tumor-promoting effects^25^,^26^. These reports may cast doubt on the safety of a chronic administration of FGF19. However, Zhou M. *et al.* have recently described an engineered form of FGF19 (M70) in which removal of the N-terminal segment does not promote hepatocellular carcinoma formation but fully retains bile acid regulatory activity^27^. As already mentioned, FGF19 expression is stimulated by FXR thus its agonists have recently attracted a major interest as a potential therapeutic agents in chronic liver conditions^28^. These include bile acids, their analogues and non-steroidal ligands of FXR. Amongst them, obeticholic acid seems to have the best documented hepatoprotective effect in humans and proved effective not only in PBC^29^ but also in non-alcoholic steatohepatitis^30^.

In non-cirrhotic PBC patients we have found several correlations between serum FGF19 levels and laboratory/clinical parameters. Firstly, serum concentration of FGF19 showed negative correlations with albumin and hemoglobin but positive ones with levels of serum bilirubin and AST. Remarkably, we have found that both liver FGF19 mRNA expression and serum FGF19 levels were strongly related to the Mayo Risk Score for PBC – a potent model for a short-term survival probability of a patient with PBC - and correlated negatively with HRQoL parameters. Moreover in our cohort of non-cirrhotic PBC patients, liver expression of FGF19 encoding mRNA increased along with stage of fibrosis. Finally, we observed a significantly higher serum level of FGF19 in patients who did not respond to UDCA. These findings support the notion that FGF19 overexpression in patients with PBC directly correlates with severity of liver disease.

Another important and novel observation relates to the association between FGF19, bile acids species and UDCA treatment. As UDCA administration is the treatment of choice in PBC, the vast majority of patients included in our study had been treated with this agent. UDCA is a potent hydrophilic bile acid, that strongly influences serum and biliary bile acid pool, thus could potentially modulate the FXR/FGF19 signaling pathway. The primary bile acid, CDCA is a natural FXR ligand, whereas secondary bile acids, deoxycholic acid (DCA) and LCA are significantly less potent agonists, and finally UDCA seems to lack FXR induction activity. However there is no data in the literature analyzing in detail the relationship between FGF19 and single bile acids species. In our previous study, in which we have investigated UDCA treatment withdrawal in patients with primary sclerosing cholangitis, serum FGF19 correlated with CDCA and CA, but only after UDCA removal, whereas UDCA showed no correlation with FGF19 levels^31^. In the current study the expression of FGF19 and FGFR4 mRNA in the examined non-cirrhotic livers did not differ between UDCA naïve and treated patients. However, circulating FGF19 levels positively correlated with taurine and glycine conjugates of CDCA, DCA and also UDCA. Moreover, patients who did not respond to UDCA had high concentrations of serum FGF19 and had higher accumulations of more hydrophilic species of bile acids in serum. To what degree a higher concentration of bile acids in serum of UDCA non-responders is related to dysfunction of bile acids transporters is an important area for further research.

In this study the CYP7A1 protein level was markedly reduced which indicates an adaptive decline of bile salts synthesis in cholestatic liver. To investigate whether the observed CYP7A1 inhibition is related to FGF19 action, we examined the involvement of MAPK- depending signaling pathways induced by activated FGFR4 which has been postulated to mediate bile acid inhibition of CYP7A1. We did not detect activation of either c-Jun or ERK1/2 kinase in cholestatic tissue. These findings may suggest that in chronic cholestasis the suppression of CYP7A1 may be additionally regulated by an alternative mechanism such as hepatic FXR/SHP signaling pathway, which is independent of the FGF19/FGFR4 axis. Studies in mice demonstrate that bile acid-activated FXR induces the expression of a negative nuclear receptor, SHP which is involved in the downregulation of CYP7A1 gene transcription. In fact, we observed a significant increase of SHP protein in cholestatic livers which was accompanied by enhanced expression of FXR, the main transcription factor involved in induction of SHP transcription. These data suggest that the observed suppression of CYP7A1 in cholestatic livers may be related to FXR-SHP signaling transduction. Thus our study implied the involvement of two parallel regulatory pathways of CYP7A1 which are independent of enterocyte-derived FGF19, to be precise, one is governed by the hepatic FXR/SHP axis and the second one acts via, a not yet defined, paracrine/autocrine action of hepatic FGF19.

We would like to comment on some limitations of our data. First of all, this is a clinical study which does not allow us to define the exact molecular mechanisms behind the regulation of FGF19 expression in patients with PBC. Analyzed factors play a pleiotropic role in a network of diverse signaling pathways, thus observed changes in their expression should be interpreted in the view of the combined effect of coexisting regulatory mechanisms. Moreover, expression of FGF19 *in vivo* is influenced by several factors which potentially may affect results.

In conclusion we have shown for the first time that liver expression of FGF19 is increased in PBC. Whether this upregulation reflects the contribution of FGF19 to compensatory mechanisms activated in this condition requires further investigation. FGF19 also correlates with severity of liver disease measured both by laboratory parameters and Mayo Risk score and is associated with impaired HRQoL. We therefore speculate that FGF19 could potentially serve as a valuable indicator of cholestatic liver injury in clinical hepatology.

## Material and Methods

### Patients

Two groups of patients in whom the diagnosis of PBC was established on the grounds of the EASL criteria^32^ were included in this study.

### Group 1 – analysis of FGF19 in serum

Fifty one patients: 45 (88.2%) females and 6 (11.8%) males with PBC, aged 60.4 ± 11.1 years were enrolled. The disease was diagnosed at least 1 year before study enrollment (mean 3.5 ± 4.0 years). Twenty three (45.1%) patients had liver cirrhosis confirmed with either histology or imaging study. Forty seven (92.2%) were treated with ursodeoxycholic acid (UDCA, range 10-15 mg/kg b.w. for the duration of at least 12 months). Twenty one (41.2%) patients were UDCA responders, 18 (35.3%) – UDCA non-responders. In 12 patients UDCA response data were not available. Clinical data, Mayo Risk Score for PBC, HRQoL questionnaires and fasting blood samples for liver biochemistry, FGF19 and bile acids were analyzed in this group. Demographic and clinical data are summarized in Table 2.

### Group 2 – analysis of FGF19 in liver tissue

Tissues from non-cirrhotic and cirrhotic PBC livers were collected. Non-cirrhotic PBC group contains 24 female patients aged 51.6 ± 10.9 years who underwent liver biopsy. Typical histological features of PBC were seen in each biopsy with following stages of fibrosis: stage 1 in 9 (37.5%), stage 2 in 7 (29.2%) and stage 3 in 4 (16.7%) biopsies. In 4 (16.7%) liver tissues there was no fibrosis. Nine (37.5%) patients from this group received a standard treatment with UDCA at the time of liver biopsy. Demographic and laboratory data on liver biochemistry and Mayo Risk Score for PBC as well as HRQoL questionnaires were collected at the time of biopsy. End-stage cirrhotic liver tissues were obtained from 21 patients (17 females, 4 males) who underwent liver transplantation. All patients were treated with UDCA at the time of surgery. Demographic and clinical data on these patients are summarized in Table 4. Control liver tissues (n = 21) were obtained from large margin liver resections of colorectal metastasis from patients with normal liver biochemistry. They were acquired from histologically normal liver, confirmed by an experienced pathologist, localized distant from the tumor^33^,^34^.

### Serum FGF19 and biochemical assays

Serum FGF19 levels were measured with a sandwich enzyme-linked immunosorbent assay -FGF19 QuantikineH ELISA kit (R&D Systems, USA), following the manufacturer’s instructions as described elsewhere^31^. Biochemical parameters including cholestatic parameters, were measured by standard laboratory methods.

### Serum bile acids determination

Blood samples for bile acids determination were collected in EDTA-tubes. Immediately after collection, plasma was purified through centrifugation at 4 °C. Subsequently one volume of formic acid 0.5 M was added to plasma samples. Samples have then been frozen and kept at −80 °C until analyses. Bile acids were purchased from Steraloids (Newport, RI), while glucuronidated acids were produced as reported^35^. Deuterated isotopes used as analytical standards were from C/D/N Isotopes, Inc. (Pointe-Claire, Québec, Canada). For bile acid-glucuronide metabolites, deuterated standards were synthesized as described^35^. All chemicals and solvents were of highest grade. Methanol, ethyl-acetate, hexane, isooctane, 1-chlorobutane, and isoamyl alcohol were obtained from VWR (Montréal, Québec, Canada). Pyridine was purchased from Regis Technologies (Morton Grove, IL). Ammonium hydroxide, citric acid, and acetic acid were obtained from Fisher Scientific (Ottawa, Ontario, Canada). Other reagents were purchased from Sigma Aldrich Co (Oakville, Ontario, Canada). Solide phase extraction columns were from Phenomenex (Torrance, CA), Varan Inc. (Palo Alto, CA) or Waters (Milford, MA). Unconjugated, taurine-, glycine- sulfate-, and glucuronide-conjugated bile acid levels measured by validated liquid chromatography/tandem mass spectrometry methods^35^,^36^,^37^,^38^. The lower limit of quantification varied from 1.0 (LCA-3G) to 8.0 nM (TCDCA) as reported^35^,^36^,^37^,^38^.

### Liver tissue preparation

Tissue samples extracted from explanted cirrhotic livers were immediately frozen in liquid nitrogen and stored at −75 °C. Specimens from non-cirrhotic livers were obtained by percutaneous needle liver biopsy. One part of it was stored in RNA*later* (Applied Biosystems, USA) while the second was immersed in formalin and later embedded in paraffin. Histological assessments of liver tissue from non-cirrhotic PBC patients were performed by a pathologist who was blinded to the clinical and laboratory data.

### RNA extraction and quantification of gene expression using Real-Time PCR

Total RNA was extracted from liver tissue using the RNeasy Mini kit (Qiagen, USA) then cDNA synthesis was carried out with the Superscript II RT kit (Invitrogen, USA). PCR reactions containing 10 μL of TaqMan® Gene Expression PCR Master Mix, 2 μl of cDNA and 1 μl of the probe/primer mix i.e. FAM-labeled probe for FGF19 or control human GAPDH (Hs00192780_m1, Hs99999905_m1; Applied Biosystems) were completed in duplicate. Quantification of gene expression was performed by means of a 7500 Fast Real-Time PCR System (Applied Biosystems) and the ΔΔCt method was used to compare the amount of target gene in experimental versus control conditions.

### Immunohistochemistry

Frozen liver sections were fixed by methanol and acetone mixture (1:1) at −20 °C for 5 minutes. Immunofluorescence analyses of the examined proteins were carried out with rabbit anti-FGFR4 (8562, Cell Signaling) and mouse anti-FGF19 (MAB969, R&D Systems) antibodies. Then incubation with either Fluorescein(FITC)-conjugated anti-rabbit IgG (711-095-152, Jackson ImmunoResearch) or Rhodamine Red^TM^-X-conjugated anti-mouse IgG (715-295-150, Jackson ImmunoResearch) was performed. Vectrashield Mounting Medium with DAPI (H-1200, VECTOR) were used to envision cell nuclei. The negative controls, in which the primary antibodies were omitted, were included in the study (data not shown). Additionally, liver tissue structures were visualized by Mayer’s Hematoxylin staining (DAKO). Images were acquired with ZEISS Axio Imager Z2 fluorescence microscope equipped with Zen Pro 2011 acquisition program.

### Western Blot Analysis

Frozen liver tissues were lysed in a RIPA buffer supplemented with protease inhibitor cocktail (Complete Mini Tablets, Roche) and phosphatase inhibitors (PhosSTOP EASYpack, Roche). Sixty micrograms of protein were resolved in SDS polyacrylamide gels and transferred onto a PVDF membrane using semi-dry transfer (Immobilon-P, Millipore). Primary antibodies were as follows: anti-FGF19 (MAB969, R&D Systems), anti-FGFR4 (8562; Cell Signalling), anti-CYP7A1 (sc-25536, Santa Cruz), anti-SHP (sc-15283, Santa Cruz), anti-ERK1/2 (4695S; Cell Signalling), anti-P-ERK1/2 (9101S; Cell Signalling), anti-cJun (9165, Cell Signaling), anti- P-cJun (2361, Cell Signaling). Protein loading was normalized to α/β tubulin (2148; Cell Signalling) or to β-actin (sc-47778; Santa Cruz). The bands were visualized with SuperSignal West Pico Chemiluminescent detection system, (Thermo Scientific). Image and densitometry analyses were performed with MicroChemi Imaging Systems and GelQuant software (DNR Bio-Imaging, Israel).

### Assessment of quality of life

The HRQoL was assessed in group 1 and non-cirrhotic subjects from group 2 with both generic (The Medical Outcomes Study Short Form-36, SF-36) and disease-specific questionnaires (PBC-40). The PBC-40 was constructed for evaluation of the HRQoL in patients with PBC^39^. It contains 40 questions in following domains: *Fatigue, Cognitive, Social-Emotional, Itch* and *Other Symptoms* with higher scores indicating poorer HRQoL. PBC-27 is a simplified questionnaire for PBC, which appears to be equally appropriate in detecting the impact of PBC on patients’ well-being^40^.

SF-36 is a widely used and validated HRQoL questionnaire, which includes 36 items divided into 8 scales. Scores can be obtained for each scale or can be aggregated into 2 summary scores, a *Mental Component Summary* and a *Physical Component Summary* score. Scale scores range between 0 and 100, with the higher score indicating better HRQoL^41^.

### Ethics

Written informed consent was obtained from each patient included in the study. The study protocol was approved by the Ethics Committee of Pomeranian Medical University and conforms to the ethical guidelines of the 1975 Declaration of Helsinki (6th revision, 2008).

### Statistics

Data were evaluated as mean ± standard deviation (SD) for continuous variables. Data were analyzed using Stat-View-5 Software (SAS Institute, Cary, NC, US) and included Fisher’s exact and ANOVA analysis. Categorical data were compared using Levene’s test for equality of variances, and both pooled-variances and separate-variances t-tests for equality of means. Correlation analysis was performed using the Spearman rank or Pearson’s correlation method. To identify independent relationships and adjust the effects of covariates, multiple linear regression analyses were performed including all parameters with highly significant correlations in the univariate analysis as covariates. A *p* value <0.05 was considered statistically significant.

## Additional Information

**How to cite this article**: Wunsch, E. *et al.* Expression of hepatic Fibroblast Growth Factor 19 is enhanced in Primary Biliary Cirrhosis and correlates with severity of the disease. *Sci. Rep.* **5**, 13462; doi: 10.1038/srep13462 (2015).

## Figures and Tables

**Table 1:** Correlations between serum bile acids concentration and FGF19.

**Bile acid species**	**Correlation coefficient (r)**	***p*** **value**
UDCA	0.146	0.30
**GUDCA**	**0.586**	**<0.0001**
**TUDCA**	**0.749**	**<0.0001**
LCA	−0.055	0.70
GLCA	−0.111	0.44
TLCA	−0.030	0.83
LCA-S	0.062	0.66
**LCA-3G**	**0.493**	**0.0002**
LCA-24G	-0.152	0.27
CA	0.041	0.77
**GCA**	**0.679**	**<0.0001**
**TCA**	**0.311**	**0.03**
CA-24G	0.017	0.90
CDCA	0.024	0.46
DCA-3G	−0.027	0.85
DCA-24G	−0.143	0.32
**GCDCA**	**0.674**	**<0.0001**
**TCDCA**	**0.370**	**0.008**
**CDCA-3G**	**0.442**	**0.001**
CDCA-24G	0.049	0.73
DCA	−0.064	0.66
GDCA	0.061	0.67
TDCA	0.031	0.83
HDCA	−0.105	0.46
HDCA-6G	−0.084	0.56
HDCA-24G	0.116	0.42
HCA	0.031	0.83
HCA-6G	−0.064	0.66
HCA-24G	−0.064	0.65
**Total bile acids**	**0.663**	**<0.0001**

Abbreviations: UDCA, GUDCA, TUDCA – Ursodeoxycholic acid and its glycine and taurine conjugates, respectively; LCA, GLCA, TLCA, LCA-3G, LCA-24G – lithocholic acid, and its glycine, taurine, 3-O-glucoronide and 24-O-glucuronide conjugates, respectively; LCA-S – sulpholithocholate, CA, GCA, TCA, CA-24G – cholic acid and its glycine, taurine and 24-O-glucuronide conjugates, respectively; CDCA, GDCA, TGCDA, CDCA-3G, CDCA-24G – chenodeoxycholic acid and its glycine, taurine, 3-O-glucoronide and 24-O-glucuronide conjugates, respectively; DCA, GDCA, TDCA, DCA-3G, DCA-24G – deoxycholic acid and its glycine, taurine, 3-O-glucuronide and 24-O-glucuronide conjugates, respectively; HDCA, HDCA-6G, HDCA-24G – hyodeoxycholic acid and its 6-O-glucuronide and 24-O-glucuronide conjugates, HCA, HCA-6G, HCA-24G – hyocholic acid and its 6-O-glucuronide and 24-O-glucuronide conjugates.

**Table 2:** Clinical and laboratory characteristics of study group 1.

**Feature**	**All patients (n** **=** **51)**	**UDCA responders**^a^ **(n = 21)**	**UDCA non-responders**^a^ **(n = 18)**	***p* value^b^**
Age (years)	60.4 ± 11.1	62.2 ± 14.3	60.3 ± 9.1	0.6
Gender (F/M)	45/6	20/1	15/3	0.43
Cirrhosis (yes/no)	23/28	8/13	12/6	0.06
Hemoglobin (mg/dl)	13.1 ± 1.5	13.7 ± 1.3	12.2 ± 1.5	**0.02**
ALT (IU/l; Normal:<30)	71.4 ± 131.0	88.6 ± 194.5	58.7 ± 37.9	0.49
AST (IU/l; Normal:<30)	60.8 ± 57.6	55.7 ± 75.2	74.7 ± 47.1	0.33
ALP (IU/l; Normal:<120)	196.7 ± 149.1	111.2 ± 44.5	278.9 ± 149.4	**0.0003**
GGT (IU/l; Normal:<42)	219.3 ± 331.8	72.8 ± 57.7	271.4 ± 232.8	0.06
Bilirubin (mg/dl; Normal:<1.0)	1.81 ± 4.5	1.1 ± 1.4	2.9 ± 6.8	0.23
Albumin (g/dl; Normal:3.8–4.4)	4.1 ± 0.5	4.3 ± 0.4	3.8 ± 0.4	**0.002**
INR (Normal: 0.8–1.2)	1.1 ± 0.3	1.1 ± 0.1	1.2 ± 0.4	0.06
Mayo Risk Score for PBC	5.4 ± 1.6	5.0 ± 1.4	6.1 ± 1.9	**0.04**
Serum FGF19 (pg/ml)	104.0 ± 152.5	67.5 ± 42.9	167.0 ± 240.3	**0.04**

Values are given as mean ± SD, unless stated otherwise.

Abbreviations: PBC-primary biliary cirrhosis, FGF19 – fibroblast growth factor 19.

^a^In 12 patients UDCA response status was not available.

^b^p values: UDCA responders vs non-responders.

**Table 3:** Correlation between liver FGF19 mRNA expression and HRQoL parameters in patients with non-cirrhotic PBC.

	**Correlation coefficient (r)**	**p value**
PBC-40		
Other symptoms	0.375	0.09
Itch	0.259	0.26
Fatigue	0.391	0.08
Cognitive	0.393	0.08
Social and emotional	0.101	0.67
PBC-27		
Symptoms	0.185	0.42
**Dryness**	**0.519**	**0.02**
Itch	0.259	0.26
Fatigue	0.345	0.12
Cognitive	0.386	0.08
Emotional	0.347	0.12
Social	−0.021	0.93
SF-36		
Physical Functioning	−0.411	0.07
Role limitation-physical	−0.320	0.17
**Bodily Pain**	**−0.531**	**0.02**
**General Health**	**−0.478**	**0.03**
Vitality	−0.345	0.14
Social Functioning	−0.430	0.07
Role limitation-emotional	−0.430	0.07
Mental Health	−0.391	0.09
**Physical Component Summary**	**−0.498**	**0.02**
**Mental Component Summary**	**−0.446**	**<0.05**

Abbreviations: FGF19 – fibroblast growth factor 19, HRQoL – health-related quality of life.

**Table 4:** Demographic and laboratory data of study group 2.

**Feature**	**non-cirrhotic PBC (n = 24)**	**cirrhotic PBC (n = 21)**
Age (years)	51.6 ± 10.9	56 ± 9
Gender (F/M)	24 (100%)/0 (0%)	17(81%)/4(19%)
UDCA treatment (yes/no)	9 (37.5%)/15 (62.5%)	21(100%)/0 (0%)
Cirrhosis (yes/no)	0 (0%)/24 (100%)	21 (100%)/0 (0%)
Liver fibrosis stage at biopsy		N/A
F≤1	13 (54.2%)	
F2	7 (29.2%)	
F3	4 (16.7%)	
F4	0 (0%)	
ALT (IU; normal: <30)	110.5 ± 148.9	no data
AST (IU/l; normal: <30)	66.1 ± 60.4	175 ± 128
ALP (IU/l; normal: <120)	250.8 ± 176.6	477 ± 296
GGT (IU/l; normal: <42)	439.6 ± 690.6	no data
Bilirubin (mg/dl; normal:<1)	1.4 ± 2.0	9.1 ± 8.2
Albumin(g/dl; normal:3.8–4.4)	4.0 ± 0.4	no data
PT (s; normal: 13–17)	12.7 ± 0.9	no data
Mayo Risk Score for PBC	4.7 ± 1.5	no data

Values are given as mean ± SD, unless stated otherwise.

Abbreviations: UDCA – ursodeoxycholic acid, PBC-primary biliary cirrhosis.
